# A Young Adult With Myocardial Bridging: A Case Report

**DOI:** 10.7759/cureus.41452

**Published:** 2023-07-06

**Authors:** Varinder Bansro, Marvi Gurbakhshani, Haaris Siddiq, Rajendra Shetty

**Affiliations:** 1 Internal Medicine, University of Maryland Capital Region Health, Largo, USA; 2 Cardiology, University of Maryland Capital Region Health, Largo, USA

**Keywords:** coronary artery angiography, myocardial infarction with non-obstructive coronary arteries (minoca), stable angina, myocardial bridging, cad: coronary artery disease

## Abstract

Myocardial bridging (MB) is a condition where the coronary artery is intramural instead of its natural course through the epicardium. Here, we present a case of a 25-year-old male without any medical history who presented with intermittent substernal chest discomfort on exertion. EKG was suggestive of ST changes in leads V1-V4 with right axis deviation. A left heart catheterization revealed myocardial bridging of the midportion of the left anterior descending artery. Myocardial bridging is commonly not associated with severe complications. However, it is imperative to diagnose it appropriately, especially in cases of chest pain, and provide immediate treatment to prevent mortality and morbidity.

## Introduction

Myocardial bridging (MB) is an anomaly defined as a condition when a segment of one or more coronary arteries passes through the myocardium [1]. The incidence of this condition has been reported from 1.5% to 16% of patients [2]. The left anterior descending (LAD) coronary artery and the junction of the proximal and middle thirds of the artery is the most common site reported for myocardial bridging [3]. Generally, MB is considered a benign condition, but complications like ischemia and myocardial infarction, ventricular fibrillation, atrioventricular conduction blocks, and sudden cardiac death, among others, are observed. [4]. We present a case of a young male, an excellent example of the effects of myocardial bridging. Such cases are rare, and cardiologists should be familiar with such an anomaly. They should consider myocardial bridging in any young individual without cardiovascular risk factors and minimal likelihood of atherosclerosis presenting with chest pain, whether typical or atypical.

## Case presentation

A 25-year-old male came into the emergency department after his primary care physician referred him because of an abnormal ECG. He had been having on-and-off substernal chest pain with exertion for the last several weeks associated with diaphoresis, shortness of breath, and palpitations. During the episodes, he rated the pain 5 to 6 out of 10. The pain was non-radiating. Lying down and resting alleviated the pain. He denied any nausea, vomiting, or reflux symptoms. The patient endorsed being stressed for several months while preparing for his exams. He endorsed increased caffeine and energy drink intake. He denied any substance abuse. He had a strong family history of CAD in his father and grandfather. He denied any past medical problems.

**Figure 1:**
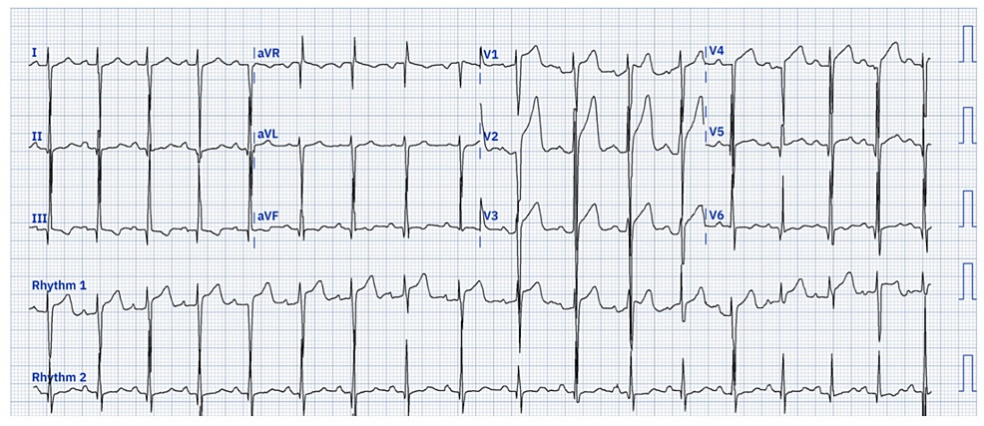
Patient’s EKG on presentation. Showing sinus tachycardia, ST changes in V1-V4, T-wave inversion in inferior leads, and right axis deviation.

Upon presentation to the emergency department (ED), his vitals were significant for heart rate 100/min sinus, blood pressure 155/96 mm Hg, respiratory rate 18/min, and SpO2 99% in room air. The physical exam was unremarkable. Troponin was < 0.010 ng/ml (normal 0.010 - 0.029 ng/ml), and BNP 22 pg/ml (normal < 100 pg/ml). The urine drug screen was negative. All other lab parameters, including complete blood count (CBC), comprehensive metabolic panel (CMP), and thyroid-stimulating hormone (TSH), were within normal limits. CXR was unremarkable. An electrocardiogram (ECG) was significant for sinus tachycardia, ST changes in V1-V4, T-wave inversion in inferior leads, and right axis deviation (Figure 1). The differential diagnosis included hypertrophic cardiomyopathy and acute MI.

**Figure 2:**
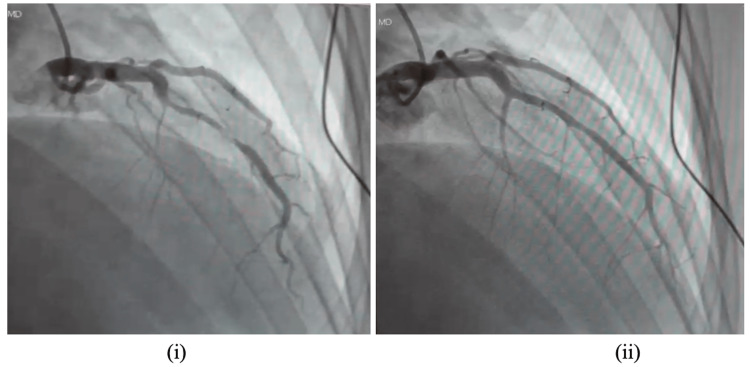
Cardiac catheterization showing myocardial bridging of the mid portion of the left anterior descending artery. (i) During Systole (ii) During Diastole

The cardiologist was consulted, who decided to activate the catheterization (cath) lab for a left heart cath. A left heart cath was done, which showed normal LVEF and normal coronaries. There was evidence of myocardial bridging in the midportion of the left anterior descending artery (Figure 2). LV pressure was 113/2 mm Hg with end-diastolic pressure of 14 mm Hg. Aortic pressure was 102/64 mm Hg. The mean arterial pressure was 79 mm Hg. Ventriculogram showed a hyperdynamic left ventricle. A transthoracic echocardiogram (TTE) was suggestive of LVEF of 64% with no significant valvular abnormalities. There were no significant wall motion abnormalities noted.

The patient was discharged on the same day with the recommendation to monitor his heart rate while exercising. He was also advised to avoid stress and anxiety. He was also advised to get a cardiac MRI to rule out structural heart disease. 

## Discussion

Commonly, the coronary arteries lie over the surface of the heart. However, in myocardial bridging (MB), one or more coronary arteries pass through the myocardium, the muscular middle layer of the heart [1]. The fold of the muscle over this artery forms a bridge, and the portion of the artery that goes through the muscle is referred to as a tunneled artery [1]. 

MB was previously considered a condition that was not dangerous or harmful. However, different studies have shown that many serious problems, such as myocardial infarction, stable or unstable angina pectoris, arrhythmias, AV blocks, and sudden cardiac death, are associated with this condition [4,5]. Moreover, it is clinically manifested that this condition is common in young adult men. Also, typical or atypical chest pain can occur at rest or on exertion [5]. The patient presented in the case above is also a young 25-year-old male with a symptom of substernal chest discomfort on exertion.

Though any coronary artery can be involved in MB, the studies showed that the left anterior descending artery is most commonly involved in MB. In contrast, the left circumflex and right coronary arteries are rarely affected [6].

Literature suggests that in young patients with a low risk for atherosclerosis or other cardiovascular diseases, if present with typical or atypical angina, MB should be suspected as it can be caused by the extra pressure on the blood vessel under the bridge when the heart muscle contracts. Thus, MB is considered a risk factor for Myocardial Infarction with Non-Obstructive Coronary Arteries (MINOCA) [7].

The tunneled coronary artery, relaxed during the diastolic phase, gets constricted during the systole phase of the cardiac cycle [8]. Consequently, the typical characteristic of an MB is systolic constriction of the coronary artery on angiography. As usually 15% of coronary blood flow occurs during systole and 85% of the coronary blood flow occurs during diastole, myocardial bridging event gains clinical significance in particular conditions, such as tachycardia. Tachycardia and exercise can induce symptoms and ischemia by decreasing the diastolic filling times and increasing the systolic phase of the cardiac cycle [9]. It is suggested in pathologic studies that lengthy and deep bridged vessels have a high risk of cardiac events [10].

Diagnostic techniques for an MB include coronary angiography, intravascular ultrasound, coronary computed tomography angiography, optical coherence tomography, and intracoronary Doppler studies [1]. Coronary angiography is a gold standard for diagnosing MB [1]. Coronary angiography was done on the presented patient, revealing MB of the left anterior descending artery's mid portion. Intravascular ultrasound helps illustrate the MB's location, diameter, and length. It also helps determine atherosclerosis's presence and distribution [1]. Due to its three-dimensional capacity and high contrast resolution, cardiac computed tomography helps identify and characterize MB. It is also valuable for determining the length and depth of MB by visualizing the lumen, the coronary artery wall, and the myocardial wall [1]. In the case of intravascular ultrasound or coronary computed tomography angiography, further testing is usually required for functional assessment of findings [1]. Optical coherence tomography can provide helpful information regarding the observation of atherosclerotic plaques and morphology of coronary arteries, while intracoronary Doppler studies are beneficial for the functional assessment of MB [1].

Patients with symptoms and signs of ischemia require treatment. Pharmacologic treatment is preferred in patients. Revascularization treatment is considered for patients whose symptoms are refractory to maximal pharmacologic therapy [1]. Beta-blockers are the first-line agents for treating symptomatic patients and act with negative chronotropic and inotropic effects. They decrease heart rate and force of contraction and reduce the constriction of the bridged segment [11]. Beta-blockers also increase the diastolic filling time. In patients intolerant to beta-blockers due to bronchospasm or in whom beta-blockers are contraindicated, calcium channel blockers can be used as they have almost similar effects on the heart [12]. Nitrates are prohibited as they cause an increase in heart rate and contraction due to reflex sympathetic triggering and thus worsen systolic constriction of the tunneled artery.

Revascularization should be considered if symptoms do not improve with maximal medical therapy. Revascularization can be with either percutaneous coronary intervention with stent placement or surgical (CABG and myotomy) intervention [1]. Further research is required to assess the best therapeutic approach in patients with myocardial bridging. The decision of the revascularization strategy also depends on the length and depth of MB [1].

## Conclusions

Patients with myocardial bridging may present exertional symptoms of myocardial ischemia, syncope, and sudden death. This case report shows myocardial bridging in a young male who presented with exertional chest pain. The case report also elucidates pathophysiology, diagnostic modalities, and treatment options for this challenging clinical entity.
